# Placental Parasitic Infections and Pregnancy Outcomes Among Women Delivering at a Tertiary Hospital in Northern Tanzania

**DOI:** 10.24248/eahrj.v6i2.695

**Published:** 2022-11-30

**Authors:** Eustadius Kamugisha Felician, Octavian Aron Ngoda, Ola Farid Jahanpour, Jackson Kahima, Sia Emmanuel Msuya, Abdul Hamid Lukambagire

**Affiliations:** a Department of Epidemiology & Biostatistics, Institute of Public Health, KCMUCo, Moshi–Tanzania; b Bukoba Regional Referral Hospital, Bukoba–Tanzania; c Tanzania Medicine and Medical Devices Authority-TMDA; d Department of Community Medicine, Kilimanjaro Christian Medical Centre, Moshi–Tanzania; e Bugando Medical Centre, Mwanza–Tanzania; f Kilimanjaro Clinical Research Institute, Moshi–Tanzania; g Department of Veterinary Medicine and Public Health, College of Veterinary Medicine and Biomedical Sciences, Sokoine University of Agriculture, Morogoro–Tanzania

## Abstract

**Background:**

Placental parasitic infections continue to be a public health problem despite numerous interventions put in place. Placental parasitic infections reported are Toxoplasma, Trypanosome, Borrelia, Schistosoma, Hookworm and Plasmodia. The infections persist to cause poor pregnancy outcomes such as maternal anaemia, low birth weight and stillbirth. This study aimed to determine the prevalence and pregnancy outcomes associated with placental parasitic infections at a tertiary hospital in northern Tanzania.

**Methods:**

A cross sectional study was conducted at Kilimanjaro Christian Medical Centre between June and July 2016. Pregnant women were interviewed before delivery and additional information obtained from their medical files. Blood samples as well as placental material were collected from each mother. Malaria was tested using a malaria rapid diagnostic test (mRDT). A total of 80 placental slide sections were made following histological protocols. After staining, slide sections were examined for the presence of parasites microscopically. Pearson's Chi-square and Fisher's exact tests were used to test for differences between groups.

**Results:**

Placental malaria parasites were found on histological examination of 8(10%) mothers' placental sections, none of whom had a positive mRDT. Education status was significantly associated with placental malaria (*p*=0.035). Stillbirth, maternal anaemia and pre-eclampsia were significantly associated with placenta malaria (*p*<0.05).

**Conclusion:**

Placental malaria was found to be prevalent in the studied population and was associated with stillbirth, maternal anaemia and pre-eclampsia. Efforts for developing malaria tests that will detect subclinical infections are needed in order to identify infections early and offer prompt treatment to prevent poor pregnant outcomes.

## BACKGROUND

Placental parasites infect millions of pregnant women in the world each year, and either directly or indirectly lead to fetal complications like intrauterine growth retardation, congenital malformations and fetal loss.^1^ There is a regional variability in the prevalence of placental parasites affecting pregnant women. In Latin America 1, 1250,000 women are infected annually with *Trypanosomacruzi.*^2^ Each year, 25 million pregnant women in sub-Saharan countries are at risk of placental infections, the majority reported are from *Plasmodium falciparum.*^3^ In Tanzania, the prevalence of placental parasitemia has been reported to range from 8%^4^ to 63.5%.^5^ In Kilimanjaro, the Malaria Survey conducted in 2017 reported the maximum monthly prevalence of placental infection in antenatal care to be ≥2% to <5%.^6^ The reported placental infections include; *Toxoplasma, Trypanosome, Borrelia, Schistosoma, Hookworm and Plasmodia.*^5^

Placental infections have been associated with maternal and neonatal mortality^7–9^ as well as morbidity.^7–11^ It is estimated that up to 200,000 deaths per year in sub-Saharan Africa result directly from placental parasitic infections.^7^Maternal anemia,^8–10^ stillbirth,^8,11^ premature delivery, intrauterine growth retardation and low birth weight^7,8,11^are among the outcomes of placental parasites.

Malaria incidence fell by 37% from 2000 to 2015 and one of the targets of the Sustainable Development Goal (SDG) number 3.3 is to end the epidemics of Acquired Immune Deficiency Syndrome (AIDS), tuberculosis, malaria and neglected tropical diseases by 2030.^12^ Successful control of placental parasites especially malaria in pregnant women is a major step towards reducing the disease burden in Africa. Control of these parasites in pregnancy involves preventing infection as well as clearing parasitemia when it occurs.^13^ Preventive measures put in place by the World Health Organization (WHO) include keeping a clean environment, use of Insecticide Treated Nets (ITN), intermittent preventive treatment in pregnancy and effective case management.^14^ The use of preventive treatment like Sulfadoxine Pyrimethamine (SP) has been shown to be effective in pregnancy.^14^

The Ministry of Health (MOH) of the Government of United Republic of Tanzania adopted SP as the preferred chemo preventive method in pregnancy with set guidelines for its use. Despite these interventions, placental parasitic infections, especially malaria, may still occur. The presence of placental receptors for parasites may enable their sequestration, which may then cause re-infections or ultimately lead to maternal and neonatal complications.^15^ However, there are limited studies in Tanzania to quantify these infections especially after the introduction of various interventions. The majority of researchers would use daily routine (eosin and hematoxylin) or Giemsa stain to investigate placental parasite infections.^16^ The use of both stains may provide more accurate findings, whereby Giemsa stain would reveal parasites like *Leishmania,*^17^ while eosin and hematoxylin would reveal malaria parasites.^18^ This study was carried out to determine the prevalence, risk factors and peripartum maternal outcomes associated with placental parasitic infections among women who delivered at Kilimanjaro Christian Medical Centre (KCMC). This information can be used to plan effective measures for reducing maternal and fetal risk factors and outcomes resulting from parasitic infections.

## METHODS

A hospital based cross sectional study recruited delivering mothers from June to July 2016 at the KCMC referral and consultant hospital serving the northern zone in Tanzania. Pregnant women aged 18 to 40 years who delivered and were admitted to the labour ward were invited to participate in this study after providing written, informed consent. Pregnant women who did not consent, had planned abortion and those that experienced miscarriage were excluded from this study. A total of 80 pregnant women met the inclusion criteria and consented to participate in this study. A non-probability convenient sampling technique was used to select study participants. A hemoglobin test using Haemoglobinometer HemoCue 201+ machine and the rapid malaria detection test (mRDT)using SD BIOLINE Malaria Antigen P.F HRP2/PLDH, followed by microscopic examination of blood slides on positive samples using Olympus CX31 Binocular Microscope, was done for each participant at enrollment. Hemoglobin test, rapid malaria detection test and microscopic examination of blood slides were performed the clinical laboratory. After delivery, the maternal surface of the placenta was washed with normal saline and then incised with a scalpel and specimens fixed in 10% neutral buffered formalin. One full placental block with 4-5μm thickness was prepared by dehydration using acetone for two hours using Semi-Automated Rotary Microtome M-240, clearing by using Xylene for two hours, and paraffin infiltration with paraffin wax. Two slides' sections were made and stained differently; one slide stained with Hematoxylin and Eosin (H&E) as the routine stain and other slide stained with Giemsa stain as the special stain. The slides were then examined by light microscopy. Placental parasites were recorded during examination and all diagnoses confirmed by an experienced pathologist at Bugando Medical Centre (BMC) pathology laboratory for External Quality Control (EQC). Pre-tested questionnaires were used to collect socio demographic information where examination record files and clinic card were used to collect clinical characteristics.

Maternal anemia, stillbirth, low birth weight and preeclampsia were the main outcomes assessed. Low birth weight was defined as an infant born with a weight of less than 2.5kg while maternal anemia as a pregnant mother with hemoglobin level less than 11g/dl. This was further categorized into mild anemia 10.0-10.9g/dl, moderate anemia 7.0-9.9 g/dl, and severe anemia <7.0 g/dl as adapted from a study conducted in Jordan.^19^ Pre-eclampsia was defined as a blood pressure of greater than 140/90 and protein in urine. Stillbirth, the death of an infant before delivery in a term pregnancy, was based on the first day after the mother's last menstrual period. The exposures for placental parasitic infections included maternal age, maternal occupation, marital status, area of residence; types of diet often used (nutrition), consumption of soil, education status and gravidity. Analysis was conducted using SPSS Inc. Released 2009. PASW Statistics for Windows, Version 18.0. Chicago: SPSS Inc. Pearson's Chi-square test was used to determine the association between the exposures and outcomes of interest. A *p value* of less than 0.05 was considered statistically significant.

### Ethics Approval and Consent to Participate

Approval to conduct this study was obtained from the Kilimanjaro Christian Medical College Research and Ethical Review Committee (CRERC) with ethical clearance certificate code number 2103, an independent review board for the medical college. Pregnant women aged 18-40 years who were admitted to the labor ward and delivered were invited to participate in this study after providing written, informed consent. Individual level medical information obtained from those mothers before and after delivery was kept strictly confidential.

## RESULTS

The socio-demographic characteristics of the pregnant mothers are summarized in Table 1. The median age of the 80 participants was 32 years (IQR 24-36). Most mothers had secondary or higher education 53(66.2%) and were multigravida 51(63.8%). Proportion of anemia cases was 23(28.7%).

**TABLE 1:** Socio-Demographic Characteristics of Women whose Placenta were Examined for Parasitic Infections (N=80)

Characteristics	Frequency	Percentage
Education		
Primary education	27	33.8
Secondary education	43	53.7
Higher education	10	12.5
Age(year)		
≤24	22	27.5
>24	58	72.5
Gravidity		
Primigravida	29	6.2
Multigravida	51	63.8
Birth weight (Kg)		
<2.5	20	25.0
≥2.5	60	75.0
Gestation age (Week)		
28-36	36	45.0
37-42	44	55.0
Hemoglobin level/anemia (g/dl)		
Normal (> 11)	57	71.3
Moderate (9.0 - 10.9)	9	11.2
Mild (7.0 - < 9.0)	14	17.5
Severe (< 7.0)	0	0
Occupation		
Employed	43	53.8
Self-employed	37	46.2
Soil Consumption		
Yes	55	68.8
No	25	31.2

Key: Kg — Kilogram; g/dl — grams/deciliter; χ^2^— Chi-square

Histopathological examination revealed that 8(10%) of placenta were infected with malaria parasites, despite the fact all mothers had negative mRDTs test results. Placental malaria infection was significantly associated with level of education of the mother (Table 2).

**TABLE 2:** Association Between Socio-demographic Characteristics and Parasitic Infections

Characteristics	Number	Parasitic Infections		χ^2^ (p-value)
		Seen (%)	Not seen (%)	
Occupational				2.956 (0.135)
Employed	43	2 (4.7)	41 (95.3)	
Self employed	37	6 (16.2)	31 (83.8)	
Pica habit				1.455 (0.424)
Yes	55	7 (12.7)	48 (87.3)	
No	25	1 (4.0)	24 (96.0)	
Education				6.960 (0.035)
Primary education	27	6 (22.2)	21 (77.8)	
Secondary education	43	2 (4.7)	41 (95.3)	
Higher education	10	0 (0.0)	10 (100.0)	
Gravidity				2.170 (0.247)
Primigravida	29	1 (3.4)	28 (96.6)	
Multigravida	51	7 (13.7)	44 (86.3)	
Gestation Age (Week)				1.437 (0.284)
28-36	36	2 (5.6)	34 (94.4)	
37-43	44	6 (13.6)	38 (86.4)	
Marital status				1.725 (0.341)
Union	67	8 (11.9)	59 (88.1)	
Non union	13	0 (0.0)	13 (100.0)	

Prevalence of placental malaria infection was associated with low hemoglobin levels (χ'=14.978, *p<0.01*), pre-eclampsia (χ'=7.485, *p=0.048*), and stillbirth (χ' =14.815, *p=.006*) (Table 3).

**TABLE 3:** Association Between Placental Malaria Parasitic Infection and Pregnancy Outcomes

Adverse pregnancy outcomes	Placenta malaria parasites			
	N	Yes n (%)	No n(%)	χ^2^ (p-value)
Still birth				14.815 (0.006)
Yes	5	3 (60.0)	2 (40.0)	
No	75	5 (6.7)	70 (93.3)	
Birth weight (Kg)				0.969 (0.459)
< 2.5	47	6 (12.8)	41 (87.2)	
≥ 2.5	33	2 (6.1)	31 (93.9)	
Hemoglobin level (g/dl)				14.978 (<0.0001)
Normal	57	1 (1.8)	56 (98.2)	
Anemia	23	7 (30.4)	16 (69.6)	
Pre-eclampsia				7.485 (0.048)
Yes	4	2 (50.0)	2 (50.0)	
No	76	6 (7.9)	70 (92.1)	

Key: Kg — Kilogram; g/dl — grams/deciliter; χ^2^-Chi-square

## DISCUSSION

Malaria was the only placental parasitic infection observed among delivering mothers in this study population. Malaria positivity among women who delivered at KCMC referral hospital was significantly associated with level of education, stillbirth, anemia and pre-eclampsia.

The prevalence observed in this study is higher than the 8% prevalence reported in the previous study^20^ and lower than 16.4% reported in another study conducted in Tanzania.^5^ Variations in community acquired immunity, sociodemographic characteristics of the study population, as well as endemicity of parasitemia which can be attributed to behavioral and environmental exposure to malaria may explain the observed differences.^21^

Additionally, geographical location, stage of pregnancy and the methods used to determine presence of parasites in the placenta may have also contribute to the observed difference between the current and previous studies. Dar es Salaam and Mwanza (high endemic area) are hotter than Kilimanjaro (low endemic area). Hotter weather has been found to favor the sporulation of Oocyst.^22^ A study in Dar es Salaam used immunosorbent agglutination assay, in Mwanza ELISA^23^ was used and in this study, manual histological staining was used.

Furthermore, the presence of placental malaria infections among participants was different based on their level of education while this difference was not seen with other socio-demographic characteristics. This revealed that pregnant women who are in the primary level of education were more likely than those with secondary and higher education to have placental malaria infection.

In this study, placental malaria was associated with anemia. Anemia is the most common consequence of *P. falciparum* malaria infection. A study conducted in Tanzania found that malaria infection during pregnancy contributed 15% of maternal anemia.^24^ The result of this outcome also corresponds with previous studies conducted in Sub-Saharan Africa.^25^ The pathogenesis of anemia by malaria parasites (*P. falciparum*) includes the hemolysis of the infected red blood cells. This is thought to be due to the reduced production of red blood cells, rupture of infected red blood cells and the destruction of uninfected cells due to antibody sensitization and with the resulting pathological effects. Marrow hypoplasia occurs in acute infections of malaria which may reduce the production of red blood cells.^21,26^ The mentioned processes may also explain how placenta malaria is significantly associated with anemia in this study.

The study also showed an association between placental malaria and stillbirth. Eight (8) percent of stillbirths worldwide (208,906) were estimated to be contributed by malaria parasites especially *P. falciparum* in pregnancy.^27^ Most stillbirths, however, occur where malaria transmission is low^3^ and the effect of malaria on stillbirth is likely to be greater in areas of low transmission where there is little or no maternal immunity.^28^ In Moshi, falciparum malaria detected at delivery, even at sub-microscopic levels may increase the risk of stillbirth. These findings suggest that even low-level, asymptomatic and/or sub-microscopic infections that might easily be missed during routine antenatal care could be detrimental to the developing fetus.^28^

Placental malaria also was significantly associated with pre-eclampsia. Seasonal changes in the incidence of pre-eclampsia have been described in tropics, which are consistent with malaria transmission periods. Placental malaria is likely to impair placental development and cause maternal hypertension and placental vascular dysfunction.^29–32^

### Strength and limitations of the study

This study used a standard histological examination, where this method can diagnose parasites which could not be detected by the rapid test (mRDT). Information was collected with the help of midwives who interviewed the women using a standardized questionnaire. To ensure data completeness and accuracy, information obtained from clinic cards was triangulated with responses from the questionnaire.

This study also had some limitations which are important to be taken into account while interpreting theresults. Firstly, the sample size was small and therefore limits the power of the inference being made. Secondly, the method used, although highly specific, can miss some other parasite as compared with more sensitive techniques like immunosorbent assay e.g. ELISA.

## CONCLUSION

These findings add to the evidence of adverse the health outcomes of placental parasitic infections among delivering mothers. Malaria in pregnancy was found to be significant associated with anemia, stillbirth as well as contributing to increased risk of pre-eclampsia. There is a need for more sensitive tests forearly diagnosis and adequate treatment during pregnancy to prevent adverse pregnancy outcomes caused by submicroscopic malaria infection.
